# Extracellular vesicles from mesenchymal stem cells alter gut microbiota and improve neuroinflammation and motor impairment in rats with mild liver damage

**DOI:** 10.1016/j.neurot.2024.e00445

**Published:** 2024-09-05

**Authors:** Gergana Mincheva, Vicente Felipo, Victoria Moreno-Manzano, Alfonso Benítez-Páez, Marta Llansola

**Affiliations:** a Laboratory of Neurobiology, Centro de Investigación Principe Felipe, Valencia, Spain; b Neuronal and Tissue Regeneration Laboratory, Centro Investigación Príncipe Felipe, Valencia, Spain; c Host-Microbe Interactions in Metabolic Health Laboratory, Centro de Investigación Principe Felipe, Valencia, Spain; d Microbiome, Nutrition and Health Research Unit, Institute of Agrochemistry and Food Technology (IATA-CSIC). Paterna-Valencia, Spain.

**Keywords:** Extracellular vesicles, Motor impairment, Kynurenine, *Bacteroides*, TNFα, Steatotic liver disease

## Abstract

Gut microbiota perturbation and motor dysfunction have been reported in steatosis patients. Rats with mild liver damage (MLD) show motor dysfunction mediated by neuroinflammation and altered GABAergic neurotransmission in the cerebellum. The extracellular vesicles (EV) from mesenchymal stem cells (MSC) have emerged as a promising therapeutic proxy whose molecular basis relies partly upon TGFβ action. This study aimed to assess if MSC-EVs improve motor dysfunction in rats with mild liver damage and analyze underlying mechanisms, including the role of TGFβ, cerebellar neuroinflammation and gut microbiota. MLD in rats was induced by carbon tetrachloride administration and EVs from normal (C-EVs) or TGFβ-siRNA treated MSCs (T-EV) were injected. Motor coordination, locomotor gait, neuroinflammation and TNF-α-activated pathways modulating GABAergic neurotransmission in the cerebellum, microbiota composition in feces and microbial-derived metabolites in plasma were analyzed. C-EVs reduced glial and TNFα-P2X4-BDNF-TrkB pathway activation restoring GABAergic neurotransmission in the cerebellum and improving motor coordination and all the altered gait parameters. T-EVs also improved motor coordination and some gait parameters, but the mechanisms involved differed from those of C-EVs. MLD rats showed increased content of some *Bacteroides* species in feces, correlating with decreased kynurenine aside from motor alterations. These alterations were all normalized by C-EVs, whereas T-EVs only restored kynurenine levels. Our results support the value of MSC-EVs on improving motor dysfunction in MLD and unveil a possible mechanism by which altered microbiota may contribute to neuroinflammation and motor impairment. Some of the underlying mechanisms are TGFβ-dependent.

## Graphical abstract

**Figure:**
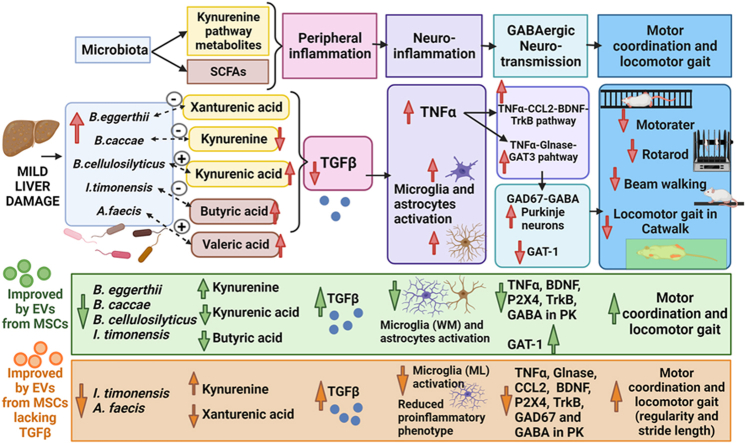

## Introduction

Obesity and metabolic syndrome may lead to steatotic liver disease (SLD), previously known as non-alcoholic fatty liver disease (NAFLD). SLD has become a significant cause of chronic liver disease. SLD progresses from steatosis to different grades of steatohepatitis and may progress to cirrhosis. A relevant percentage of patients with SLD show mild cognitive and motor impairment, which reduces their quality of life [[1], [2], [3], [4]]. The animal model of SLD comprises rats injected with CCl_4_, where liver damage also progresses from steatosis to different grades of steatohepatitis, reaching liver cirrhosis at around 12 weeks [5,6]. Rats injected with CCl_4_ also show hepatic encephalopathy, with cognitive and motor impairment [7,8]. On the other hand, rats with hyperammonemia and hepatic encephalopathy also show cognitive and motor impairment due to altered neurotransmission, which, in turn, is a consequence of neuroinflammation [9,10]. This last feature of the morbid condition altering brain function is also reproduced in patients who died with steatohepatitis or with cirrhosis and minimal hepatic encephalopathy (MHE), which show neuroinflammation in the cerebellum with activation of microglia and astrocytes [11,12], which may contribute to the motor alterations.

Beyond brain functions altered in chronic liver diseases and MHE, changes in the gut microbiota are also described as likely being involved in the induction of such maladies. Gut microbiome alterations are already known to contribute to the development of liver steatosis and cirrhosis [[13], [14], [15]]. For instance, microbiota changes in MHE patients are associated with impaired cognition, endotoxemia, and inflammation [16,17]. Moreover, in cirrhotic patients, MHE is reversed by treatment with rifaximin, a non-permeable antibiotic which acts on gut microbiota [18]. The above effects of rifaximin have also been observed in pre-clinical studies where cognitive and motor function in rats with mild liver damage induced by injection of CCl_4_ were restored upon its oral administration [7,8]. All in all, the above evidence suggests that changes in gut microbiota mediate the induction of MHE and that reversing the changes in gut microbiota may reverse cognitive and motor impairment. This notion is further supported by reports from Bajaj et al. [16,17,19] showing that gut microbiome is altered in cirrhotic patients with MHE and that fecal transplant may improve cognitive function in these patients [19]. Similarly, Liu et al. [20] propose that neuroinflammation in murine cirrhosis depends on the gut microbiome, which could be attenuated by fecal transplant. Alterations in the microbiota and the gut-brain axis also seem to be involved in the pathogenesis of neuroinflammatory and neurodegenerative diseases such as Parkinson's, Alzheimer's, and multiple sclerosis [21,22]. These reports suggest that alterations in gut microbiota could also contribute to neuroinflammation and motor impairment in rats with mild liver failure and MHE due to CCl_4_ injection.

From the epidemiology point of view, the prevalence of SLD in the general population is estimated at 24%, and it is increasing due to obesity, insulin resistance, diabetes, and metabolic syndrome [23]. Concomitantly, the prevalence of mild cognitive impairment in patients with SLD reaches approximately 32% [3], indicating that a large proportion of the human population around the world would suffer mild cognitive impairment due to SLD, thus reducing their quality of life and ability to perform daily tasks. Therefore, it is necessary to have an effective treatment that reverses cognitive and motor impairment in patients with SLD. Unfortunately, up to date, there are no specific or conclusive treatments to restore neurological function in patients with SLD. Notwithstanding, there is increasing evidence supporting the role of extracellular vesicles (EVs) from mesenchymal stem cells (MSCs) in improving neuroinflammation and cognitive and motor function in different pathologies associated with chronic inflammation [24,25]. The EVs are carriers of proteins, RNAs and miRNAs, facilitating and mediating their release inside the target cells. The EVs from MSCs reduce neuroinflammation and improve neurological outcomes in animal models of traumatic brain injury, Alzheimer's and Parkinson's disease, hyperammonemia, and MHE [[26], [27], [28], [29], [30], [31]]. Consequently, this work aimed to assess the therapeutic prospect of EVs from MSCs to revert neuroinflammation and changes in neurotransmission in the cerebellum and impairment of motor coordination and locomotor gait in a preclinical appraisal using a rat model of MHE via CCl_4_ injection. We also sought to assess if the amelioration of such neurological parameters of brain function is related to some extent to changes and composition of the gut microbiota.

## Materials and Methods

### Animal model and treatments

Male Wistar rats (Charles River) weighing 150–180 ​g were used. Rats were maintained at standard temperature conditions (22 ​°C), humidity (40–60%), and the light-dark cycle of 12:12 ​h, with a standard diet and food and drink ad libitum. To induce liver damage, rats were intraperitoneally injected three times/week for four weeks with 1 ​mL/kg body weight of carbon tetrachloride (CCl_4_). CCl_4_ was prepared 1:10 (v:v) in corn oil, as previously described by Balzano et al. [8]. Control rats were intraperitoneally injected with 1 ​mL/kg of corn oil. Rats were treated with extracellular vesicles (EV) from mesenchymal stem cells (MSC), with EVs lacking TGFβ or PBS (phosphate-buffered saline). EVs were isolated from the culture media of sub-confluent human adipocyte-derived mesenchymal stem cells (MSCs) and from a TGFβ knockdown MSCs culture through several centrifugations, as detailed previously [32]. EVs were administered twice (at two and three weeks of CCl_4_ treatment) as an intravenous injection at 50 ​μg in 300 ​μL of PBS. Control rats were injected with PBS. The experiment consisted, therefore, of 6 groups of rats: Control-vehicle (CV); CCl_4_-induced liver damage (CC); Control rats treated with MSCs-EVs (CE); Control rats treated with EVs from TGFβ knockdown MSCs (CE-T); CCl_4_ rats treated with MSC-EVs (CCE) and CCl_4_-injected rats with TGFβ knockdown MSC-EVs (CCE-T). The experiments were approved by the Ethics Committee of Animal Experimentation (*Comité de Experimentación y Bienestar Animal, CEBA*) of Centro de Investigación Príncipe Felipe (CIPF; Valencia, Spain) and the *Conselleria de Agricultura de la Generalitat Valenciana* (Valencian Community, Spain) and they were performed following the guidelines of the Directive of the European Commission (2010/63/EU) for the care and handling of experimental animals. All experiments were performed according to the ARRIBE guidelines for animal experimentation.

### Analysis of locomotor gait

Catwalk™ is a video-based system for automatic gait analysis (Noldus, Wageningen, The Netherlands). Locomotor gait was analyzed in the catwalk as previously described [33]. Three trials were recorded each day for three days. Gait analysis values are the mean of nine runs. This test was performed after three weeks of CCl_4_ administration. Data were analyzed using the Catwalk analysis software (v 7.1).

### Analysis of motor coordination in the Motorater

A kinematic analysis of motor coordination was conducted using the MotoRater apparatus (TSE Systems, Germany). It consists of a horizontal ladder that the animals have to cross while being recorded from three angles. Each day, four uninterrupted runs were recorded for each rat over two days. The runs were analyzed by counting and classifying the steps as correct or wrong paw placements (slips or misses). The results are expressed as a percentage of total steps and are the mean of eight runs.

### Analysis of motor coordination in the beam walking

The beam walking test was performed. This test consists of a wood strip 20 ​mm in diameter and 1 ​m long, located 1 ​m above the ground. Rats have to cross the beam, and two observers count the number of times the rats slip off the beam. The average number of foot faults (slips) is a measure of incoordination.

### Analysis of motor coordination in the rotarod

The rotarod (Ugo Basile, Comerio, Italy) assesses the ability of rats to stay on a rotating drum with progressive acceleration. Two consecutive days before testing, each rat was placed on the rotarod at constant speed for 3 ​min. The rotarod speed was increased from 4 to 40 ​rpm over 300 ​s during the test. Motor incoordination was calculated as the mean latency to fall (2 trials) with a maximum cut-off of 600 ​s.

### Plasma extraction and analysis of TGFβ

Plasma was obtained from the saphenous vein in ethylenediaminetetraacetic acid (EDTA) vials (BD Microtainer K2E tubes) and stored at −80 ​°C. TGFβ in plasma was measured with an ELISA kit (Invitrogen 88-50680).

### Immunohistochemistry

Rats were anaesthetized with intraperitoneal injection of 1 ​mL/kg body weight of 300 ​mg/mL sodium pentobarbital and transcardially perfused with 0.9% saline followed by 4% paraformaldehyde in 0.1 ​M phosphate buffer (pH 7.4). Brains were removed and post-fixed in the same fixative solution for 24 ​h at 4 ​°C, then they were processed with a specific brain programme in a HistoCore Pearl apparatus (Leica). Five-micrometer thick, paraffin-embedded sections (5 ​μm) were cut in a microtome (Microm) and mounted on slide glasses coated for 10 ​min with gelatin (900 ​mg/L)-chromium potassium sulphate (280 ​mg/L). Tissue sections were deparaffinized for 1 ​h at 62 ​°C, rehydrated in an AutoStainer (Leica; 10 ​min oven, 2 ​× ​10 ​min xylene, 5 ​min absolute ethanol, 2 ​× ​5 ​min 96% ethanol, 5 ​min 70% ethanol) and antigen retrieved (Tris-EDTA buffer pH ​= ​9 or sodium citrate buffer pH ​= ​6, depending on antibody brand recommendation). After that, sections were incubated with 3% H_2_O_2_ (diluted from PanReac Applichem 33% H_2_O_2_ 131077.1211) for 15 ​min to block endogenous peroxidases and with 5% normal goat serum (NGS; Abcam ab7481) in PTA (0.1 ​M phosphate buffered saline (PBS) with 0.1% BSA and 0.1% Triton-X100) for 1 ​h at room temperature to block unspecific binding. Sections were incubated with primary antibodies overnight at 4 ​°C. Primary antibodies used for the study were: anti-ionized calcium binding adapter molecule 1 (IBA1) (Wako 019-19741, 1:300); anti- Glial Fibrillary Acidic Protein (GFAP) (Sigma G3893, 1:400), GAD65 (Abcam ab26113, 1:200) GAD67 (Abcam ab26116, 1:1000) and GABA (Millipore ABN131, 1:200). Tissue sections were washed and incubated with biotinylated secondary antibodies goat anti-rabbit and goat anti-mouse (Vector Laboratories, 1:200) for 1 ​h at room temperature. After that, they were incubated with Avidin Biotin Complex (ABC) (Vector Laboratories) for 30 ​min as described by the manufacturer, which contains avidin and biotinylated horse-radish peroxidase (HRP). Finally, diaminobenzidine (DAB), colorimetric substrate for HRP, was added for a maximum of 10 ​min. For this, the Abcam ready to use kit (DAB Substrate Kit ab64238) was used following manufacturer instructions: 30 ​μL DAB Chromogen solution plus 1.5 ​mL DAB Substrate solution. Mayer's hematoxylin (DAKO S3309; Ready to use) for 1 ​min was used to counterstain nuclei. Slides were then dehydrated and mounted in an AutoStainer (Leica; 30 ​s 96% ethanol, 3 ​× ​1 ​min absolute ethanol, 2 ​min xylene and mounted with DPX).

### Astrocyte and microglia activation

Tissue sections stained with IBA1 and GFAP were scanned with the Aperio Versa system (Leica Biosystems), and images were taken with ImageScope software (Leica Biosystems). Iba1 and GFAP staining were analyzed in the white matter of the cerebellum (ten random fields per animal at 40X). The reduced perimeter of microglia (due to ameboid shape) was considered as a measure of activation. The perimeter of individual Iba1+ cells was measured with ImageProPlus software. Astrocytic activation was measured as a percentage of GFAP stained area with Image J software.

### GADs and GABA quantification

The content of GABA, GAD65 and GAD67 in Purkinje neurons was quantified by staining intensity in 10 fields 20X per rat with the Image J ROI tool. The blank (mean intensity of background) was subtracted to the mean intensity of the Purkinje neurons. Results were expressed as mean intensity of the images per rat.

### Analysis of protein content by Western blotting

The rat's cerebellums were dissected and stored at −80 ​°C. Cerebellums were homogenized in 50 ​mM TRIS–HCl pH 7.5, 50 ​mM NaCl, 10 ​mM EGTA, 5 ​mM EDTA and protease (1 ​mM Phenylmethylsulfonyl fluoride (PMSF), 10 ​μg/mL Leupeptin and 4 ​μg/mL aprotinin) and phosphatase (2 ​mM sodium-pyro-phosphate, 1 ​mM NaF and 1 ​mM sodium-orto-Vanadate) inhibitors for analysis of proteins by Western blot. Total protein content was determined by the bicinchoninic acid (BCA) method. Twenty-five μg of protein were loaded in 15 or 10% acrylamide electrophoretic gels. Protein samples were separated by SDS-PAGE and transferred to polyvinylidene difluoride (PVDF) membranes. After transference, membranes were blocked by 5% bovine serum albumin (BSA) in TBST (Tris 5 ​mM, NaCl 15 ​mM, TWEEN-20 0,01%) at room temperature while stirring for 45 ​min. After washing 3 times 10 ​min each, the membrane was incubated overnight with the appropriate primary antibodies at 4 ​°C. After washing, membranes were incubated with the corresponding alkaline phosphatase conjugated secondary antibodies for 1 ​h, while balanced at room temperature. After washing secondary antibodies, membranes were incubated with substrate buffer 10 ​min at room temperature. Then, membranes were revelated by incubating them with developing solution (0’66% NBT, 0,34% BCIP, 99% substrate buffer) the required time until good visualization of coloured bands, at room temperature. The following antibodies were used: anti-TNFα (RD Systems AF-510-NA, 1:250), anti-glutaminase I (Novus NBP-1 76544, 1:1000), anti-GAT3 (Abcam ab431, 1:500), anti-BDNF (Invitrogen OSB00017W, 1:1000), anti-TrkB (Abcam ab18987, 1:500), anti-CCL2 (Proteintech 66272-1-lg, 1:2000) and anti-YM-1 (Abcam ab93934, 1:500). β-Actin (Abcam, 1:5000) or GAPDH (Millipore, 1:15,000) were used as a control for protein loading. Secondary antibodies (1:4000) were IgGs conjugated with alkaline phosphatase (Sigma, Madrid, Spain).

The images were captured using an Epson Perfection V39 scanner, and band intensities were quantified using AlphaImager 2200 software. Values were obtained from the difference between the specific band and an equivalent region corresponding to no band, as blank, and corrected by the corresponding actin or GAPDH band intensities. Final results are shown as relative level of protein in percentage respect to the controls.

### Membrane surface expression of receptors and transporters

Dissected cerebellums were put into ice-cold Krebs buffer (in mmol/L): NaCl 119, KCl 2.5, KH_2_PO_4_ 1, NaHCO_3_ 26.2, CaCl_2_ 2.5, and glucose 11, aerated with 95% O_2_ and 5% CO_2_ at pH 7.4. Transversal 400 ​μm thick slices were obtained with a Vibratome (LEICA, Vt1000s). Slices were added to tubes containing ice-cold Krebs buffer with or without 2 ​mM bis(sulfosuccinimidyl)suberate (BS3) (Pierce, Rockford, IL, USA) and incubated for 30 ​min at 4 ​°C. Cross-linking was terminated by adding 100 ​mM glycine (10 ​min, 4 ​°C). Tissue slices were homogenized by sonication for 20 ​s. Samples treated with or without BS3 were analyzed by Western blot using antibodies against P2X4 (Invitrogen PA5-41080, 1:2000), GAT1 (Abcam ab426, 1:500) and GAT3 (Abcam ab431, 1:500). The surface expression of receptor subunits was calculated as the difference between the intensity of the bands without BS3 (total protein) and with BS3 (non-membrane protein) [34].

### Quantification of microbiota-derived metabolites

The concentration of short-chain fatty acids (SCFAs) and metabolites of the Kynurenine pathway compounds in plasma was measured by LC-MS with an EXION (Shimatzu) HPLC system coupled to a mass spectrometry detection system consisting of a QTRAP 4500 triple quadrupole (AB Sciex, Ontario, Canada) equipped with electrospray ionization (ESI) ion source, controlled by the Analyst software, version 1.6.3. The sample preparation proceeded as follows: plasma samples (30 ​μL) were derivatized with 10 ​μL of 1 ​M O-benzylhydroxylamine (O-BHA) (SIGMA) and 10 ​μL of 1 ​M N-(3-Dimethylaminopropyl)-N-ethylcarbodiimide-HCl (EDC) (SIGMA) prepared in freshly prepared pyridine buffer (270 ​μL of 12.1 ​M HCl ​+ ​430 ​μL of pyridine in H2O to 5 ​mL, pH 5), in a fume hood. This derivatization mixture was agitated at 300 ​rpm for 10 ​min. Then 50 ​μL of methanol was added, and vortexed, and 300 ​μL of dichloromethane was added for extraction by agitation at 300 ​rpm for 30 ​min. Then, 100 ​μL of the organic phase was separated and evaporated at room temperature in a fume hood. The samples were reconstituted with 85 ​μL of 0.1% formic acid in H2O and 40 ​μL and injected in the HPLC under the following conditions: a Kinetex C18 100∗4.6 ​mm 2.6 column from Phenomenex, at 40 ​°C, was used. The mobile phase consisted of a two-phase gradient: 0.1% formic acid in water (A) and 0.1% formic acid in acetonitrile (B), as follows: 5–20% B 0–1 ​min, 20–50% B 1–5.5 ​min, 60% B 5.5–5.7 ​min, 80% B 6 ​min, 80% B 6.5 ​min, 5% B 6.6 ​min, 5% B 12 ​min, with a flow rate of 0.4 ​mL/min. The conditions of the mass spectrometer were: positive ionization mode, entrance potential 10, curtain gas 30, declustering potential 60 ​V, collision energy 15 ​eV, GAS1 40 and GAS2 50, 500 ​°C and 4500 ​V in multiple reaction monitoring (MRM) mode with the following transitions for the quantification of the different SCFAs: acetic acid 166.1 ​m/z ​> ​91 ​m/z; propionic acid 180.1 ​m/z ​> ​91 ​m/z; butyric acid and isobutyric acid 194.1 ​m/z ​> ​91 ​m/z; valeric acid 208 ​m/z ​> ​91 ​m/z and caproic acid 222 ​m/z ​> ​91 ​m/z. A SCFAs mixture standard curve, from 1 to 2500 ​ng/mL (10–25000 ​ng/mL of caproic acid), was prepared in H2O and derivatized and extracted as the samples to calculate SCFAs concentrations. The sample preparation for analysis of the kynurenine pathway compounds involved protein precipitation performed in 50 ​μL of plasma samples by addition of 10 ​μL of trifluoroacetic acid (SIGMA), mixture in vortex and centrifugation at 20.000 ​*g* for 15 ​min at 4 ​°C. The supernatants were separated and 40 ​μL injected in the HPLC-MS following the next conditions: Luna Omega Polar C18 (OOD-4760-AN) 100∗2.1 ​mm 3 ​μm (100 A) column from Phenomenex, at 40 ​°C, was used. The mobile phase consists of a two-phase gradient: 0.1% formic acid in water (A) and 0.1% formic acid in acetonitrile (B), as follows: 5% B 0–0.5 ​min, 5–80% B 0.5–5.0 ​min, 80% B 5.0–6.0 ​min, 80-5% B 6.0–6.1 ​min, 5% B 6.1–8 ​min, with a flow rate of 0.4 ​mL/min. ESI ion source in positive ionization mode was used with entrance potential 10, curtain gas 30, GAS1 40 and GAS2 60, 500 ​°C and 4500 ​V in multiple reaction monitoring (MRM) mode with the following conditions for each metabolite: 1) kynurenine: 209.2 ​m/z ​> ​192 and 94 ​m/z, RT 2.5 ​EP 8 CE 15 DP 90 CXP 15 2) kynurenic acid:190.1 ​m/z ​> ​144 and 116 ​m/z, RT 2.5 ​EP 10 CE 25 DP 50 CXP 10 and 3) xanthurenic acid: 206.2 ​m/z ​> ​160 and 132 ​m/z RT 2.5 ​EP 10 CE 28 DP 130 CXP 16. A standard curve from 5 to 3000 ​nM of kynurenine and from 0.5 to 300 ​nM of kynurenic and xanthurenic acids was prepared in H2O and processed as the samples to calculate metabolite concentration in samples.

### DNA extraction and amplicon-based library sequencing

The DNA from rat feces was extracted using the QIAamp® Fast DNA Stool Mini Kit (Qiagen, Hilden, Germany) according to the manufacturer's instructions with prior treatment on FastPrep-24™ 5G bead beating grinder (MP Biomedicals, Santa Ana, CA, USA) using InhibitEX Buffer (kit extraction reagent) and 0.1 ​mm diameter zirconium/silica beads. The DNA concentration was measured by Qubit 4.0 and the Qubit dsDNA HS Assay Kit (Thermo Fisher Scientific, USA). The V3–V4 hypervariable regions of the 16S ribosomal ribonucleic acid (rRNA) gene were amplified using ∼20 ​ng DNA and 25 PCR cycles consisting of the following steps: 95 ​°C for 20 ​s, 55 ​°C for 20 ​s and 72 ​°C for 20 ​s. NZYProof DNA polymerase (NZYTech, Lisbon, Portugal) and the 7-mer barcoded primers, S-D-Bact-0341-b-S-17 (TAGCCTACGGGNGGCWGCAG) and S-D-Bact-0785-a-A-21 (ACTGACTACHVGGGTATCTAATCC) targeting a wide diversity of bacterial 16S rRNA genes [35] were used for PCR. Dual-barcoded PCR products were purified from duplicate reactions with the NZYGelpure purification Kit (NZYTech, Lisbon, Portugal) and quantified through Qubit 4.0 and the Qubit dsDNA HS Assay Kit (Thermo Fisher Scientific, USA). The samples were multiplexed by combining equimolar quantities of V3–V4 amplicons (∼40 ​ng per sample) and sequenced in one lane of the Illumina MiSeq platform with 2 ​× ​300 PE configuration (CNAG, Barcelona, Spain).

### Amplicon data analysis

Raw data were delivered in fastq files and pair ends with quality filtering were assembled using *Flash* software [36]. Assessment of alpha and beta diversity analyses were performed using the Operational Taxonomic Unit (OTU)-picking approach as follows. Sample de-multiplexing was completed with sequence information from barcoded forward/reverse primers and the *SeqKit* tool [37]. After de-multiplexing and barcodes/primers removal, the chimera detection was performed with *UCHIME* algorithm [38] and the *SILVA* reference set of 16S rRNA sequences (Release 138) [39]. The OTU information was retrieved by using the *UCLUST* algorithm implemented in *USEARCH* v8.0.1623 [40]. The alpha diversity descriptors such as Chao's index, evenness, Shannon index, Simpson's reciprocal index, Dominance index, and phylogenetic distance (PD) were computed using QIIME v1.9.1 [41] from a subset of 15,000 sequences randomly selected after random shuffling (10,000X). For phylogenetic-based metrics the OTUs sequences were aligned with PyNAST algorithm and the SILVA aligned reference database. Tree topology reconstruction was retrieved with the FastTree algorithm [42] using the generalized time-reversible (GTR) model and gamma-based likelihood. The community structure across the sample groups was performed with the *vegan* R package through interpretative multivariate appraisal based on non-metric multidimensional scaling method - NMDS (*vegan::metaMDS* function and “bray” distance). For taxonomy assessment, we used the complete set of sequences obtained after chimera removal. Following the OTU-picking approach (*UCLUST* algorithm). Taxonomy identification for OTUs of interest was assisted by utilization of SINA aligner [43] and SILVA database (Release 138).

### Statistical analysis

GraphPad Prism software v. 9.5.0 was used for statistical analysis. Data are expressed as mean ​± ​SD. Statistical analysis was carried out using one-way ANOVA and Tukey's or Fisher's LSD multiple comparisons test. Data that did not pass the normality test (D'Agostino and Pearson or Kolmogorov–Smirnov tests) were analyzed with the Kruskal–Wallis nonparametric test followed of Dunn's test for multiple comparisons. When standard deviations (SD) were not equal, Welch's ANOVA followed of Dunnett's T3 or unpaired t with Welch's correction multiple comparisons tests was applied. A confidence level of 95% was considered as significant. The transformation of compositional microbiota-derived data was achieved by applying the centered log-ratio (clr) algorithm implemented in *CoDaSeq::codaSeq.clr* with prior execution of *zCompositions:: cmultRepl* function supporting a Bayesian-Multiplicative replacement of count zeros. Non-parametric methods such as Kruskal-Wallis and pairwise Wilcoxon Rank Sum tests (for unpaired samples) with Benjamini-Hochberg *post hoc* correction for multiple group comparison were executed across the alpha diversity descriptors. Differences in the microbial community structure were assessed by interpretative approaches using the permutation-based *vegan::adonis2* function. Differential abundance of OTUs across groups was assisted by application of Kruskal-Wallis test with Benjamini-Hochberg post hoc correction and pairwise Wilcoxon Rank Sum test. Highly divergent OTUs in terms of abundance, and associated with EVs treatment, were selected by applying estimation statistics relying on size-effect (*dabestr::dabest* R function). Correlation analysis to assess the association between microbiota and metabolite variables was completed by computing Kendall's ρ (rho) parameter (*stats::cor.test* R function). Microbiota graphs and plots were drawn using ggplot2 R package (R distribution V4.1.2.).

## Results

### Effect of EV-MSCs on amelioration of impaired motor coordination and locomotor gait

**Fig. 1:**
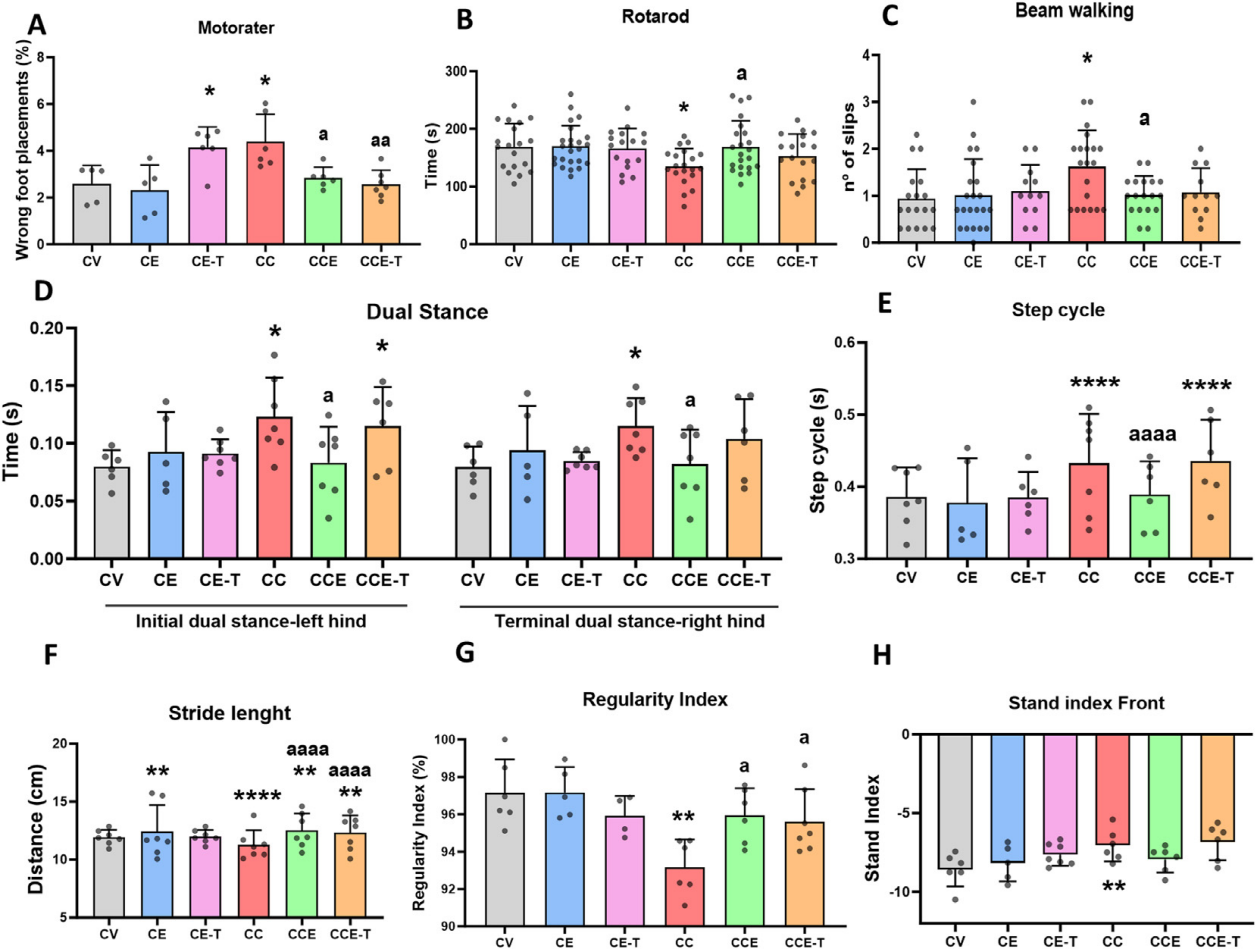
**Effects of MSCs-EVs on motor coordination and locomotor gait**. Motor function was assessed in the third week of CCl_4_ treatment. Motor coordination was assessed in the Motorater (A), the Rotarod (B), the beam walking (C) tests and the Catwalk regularity index (D). For A data are mean ​± ​SD of 5–7 rats per group and were analyzed with Kruskal-Wallis test followed by uncorrected Dunn's tests to assess the effect of C-EVs (statistic ​= ​9.893, p ​= ​0.0195) and T-EVs (statistic 13.24, p ​= ​0.0041). In B (n ​= ​17–24), C (n ​= ​12–22), and D (n ​= ​4–7) One-Way ANOVA was used: B (F ​= ​4.027, p ​= ​0.0101), and C (F ​= ​4.424, p ​= ​0.0065) the ANOVA was significant for C-EVs but not T-EVs and was followed by the Tukey's multiple comparisons test. In D, the effect of C-EVs (F ​= ​8.660, p ​= ​0.0008) was additionally analyzed by the Tukey's multiple comparisons test and the effect of T-EVs (F ​= ​6.477, p ​= ​0.0033) was followed by the Uncorrected Fisher's LSD test. Locomotor gait was assessed with some parameters from the Catwalk XT: Dual Stance (E), Stride length (F), Stand Index front (G) and Step cycle (H). Data are mean ​± ​SD of 5–7 animals per group. (E) Data were analyzed with one-way ANOVA followed by the Uncorrected Fisher's LSD test for Initial dual stance left hind (F ​= ​3.008, p ​= ​0.0456 for C-EVs and F ​= ​3.972, p ​= ​0.021 for T-EVs) and for terminal dual stance right hind (F ​= ​2.304, p ​= ​0.1063 for C-EVs and with Welch's ANOVA and unpaired t with Welch's correction, W ​= ​3.662, p ​= ​0.0510 for T-EVs) (F) one-way ANOVA (C-EVs F ​= ​50.07; T-EVs F ​= ​46.43; both p ​< ​0.0001) followed by Tukey's multiple comparisons test. (G) Welch's ANOVA (C-EVs W ​= ​127.40, p ​= ​0,0057; T-EVs W ​= ​103.8, p ​= ​0,0092) followed by Dunnett's T3 multiple comparisons test. (H) one-way ANOVA (C-EVs F ​= ​89.24; T-EVs F ​= ​101.2; both p ​< ​0.0001) followed by Tukey's multiple comparisons test. Asterisks indicate a significant difference compared with CV group ∗p ​< ​0.05, ∗∗p ​< ​0.01, ∗∗∗∗p ​< ​0.0001 and “a” compared with CC group a p ​< ​0.05, aa p ​< ​0.01 and aaaa p ​< ​0.0001.

MHE rats showed impaired motor coordination as analyzed in the different tests performed. Thus, MHE rats exhibit increased wrong foot placements (4.38 ​± ​0.48%) compared to control rats (2.59 ​± ​0.35%, p ​= ​0.0180) in the Motorater (Fig. 1A), remain less time in the rotarod (CC 135 ​± ​7 ​s, CV 169 ​± ​9 ​s, p ​= ​0.0351) (Fig. 1B) and show more slips (CC 1.62 ​± ​0.17, CV 0.94 ​± ​0.15, p ​= ​0.013) than control rats in the beam walking (Fig. 1C). MCS-EVs treatment on MHE rats reversed the impairment of motor coordination (2.84 ​± ​0.19, p ​= ​0.0184) in the Motorater (Fig. 1A), rotarod (169 ​± ​10, p ​= ​0.0274) (Fig. 1B) and beam walking (1.02 ​± ​0.10, p ​= ​0.0347) (Fig. 1C) tests, returning to values close to control rats. On the other hand, treatment of MHE rats with EVs lacking TGFβ (MSC-T-EVs) also reversed motor incoordination (2.57 ​± ​0.23, p ​= ​0.0083) in the Motorater (Fig. 1A). Concerning motor coordination assessed in the rotarod and the beam walking, MSC-T-EVs administration reversed them partially; thus, the time in the rotarod (153 ​± ​9 ​s) and the number of slips (1.08 ​± ​0.15) in the beam walking were not different in MHE rats compared to control specimens (Fig. 1B and C). We also analyzed different parameters of locomotor gait in the Catwalk. MHE rats showed increased initial dual stance in the left hind paw (0.12 ​± ​0.01, p ​= ​0.0088), terminal dual stance in the right hind paw (0.12 ​± ​0.01, p ​= ​0.02, Fig. 1D), and step cycle (0.43 ​± ​0.03, p ​< ​0.0001, Fig. 1E) and reduced stride length (11 ​± ​1, p ​< ​0.0001, Fig. 1F), regularity index (97.1 ​± ​0.7%, p ​= ​0.0014; Fig. 1G) and stand index front (−7.04 ​± ​0.42, p ​= ​0.0061; Fig. 1H). The treatment of CCl_4_-injected rats with MSC-EVs reversed the impairment of dual stance (0.08 ​± ​0.01, p ​= ​0.0202; Fig. 1D), step cycle (0.39 ​± ​0.02, p ​< ​0.0001, Fig. 1E), stride length (13.5 ​± ​0.6 ​cm, p ​< ​0.0001, Fig. 1F), regularity index (95.94 ​± ​0.60%, p ​= ​0.027; Fig. 1G), and tended to normalize stand index front; the values reached were not different from control counterparts (Fig. 1H). In a similar manner, the MSC-T-EVs also increased stride length (12.3 ​± ​0.6 ​cm, p ​< ​0.0001; Fig. 1F) and regularity index (95.62 ​± ​0.65%, p ​= ​0.0125; Fig. 1G), but did not improve dual stance (Fig. 1D), step cycle (Fig. 1E), and stand index front (Fig. 1H) in MHE animals. In general, the MSC-EVs treatment on control rats only affected stride length (Fig. 1F), showing a significant increase in the scoring (12.4 ​± ​0.9, p ​= ​0.0039); whereas MSC-T-EVs only altered the motor coordination in same control rats (4.1 ​± ​0.4, p ​= ​0.013; Fig. 1A).

### Cerebellum GABAergic neurotransmission phenotype of MHE model and effect of MSC-EVs

**Fig. 2:**
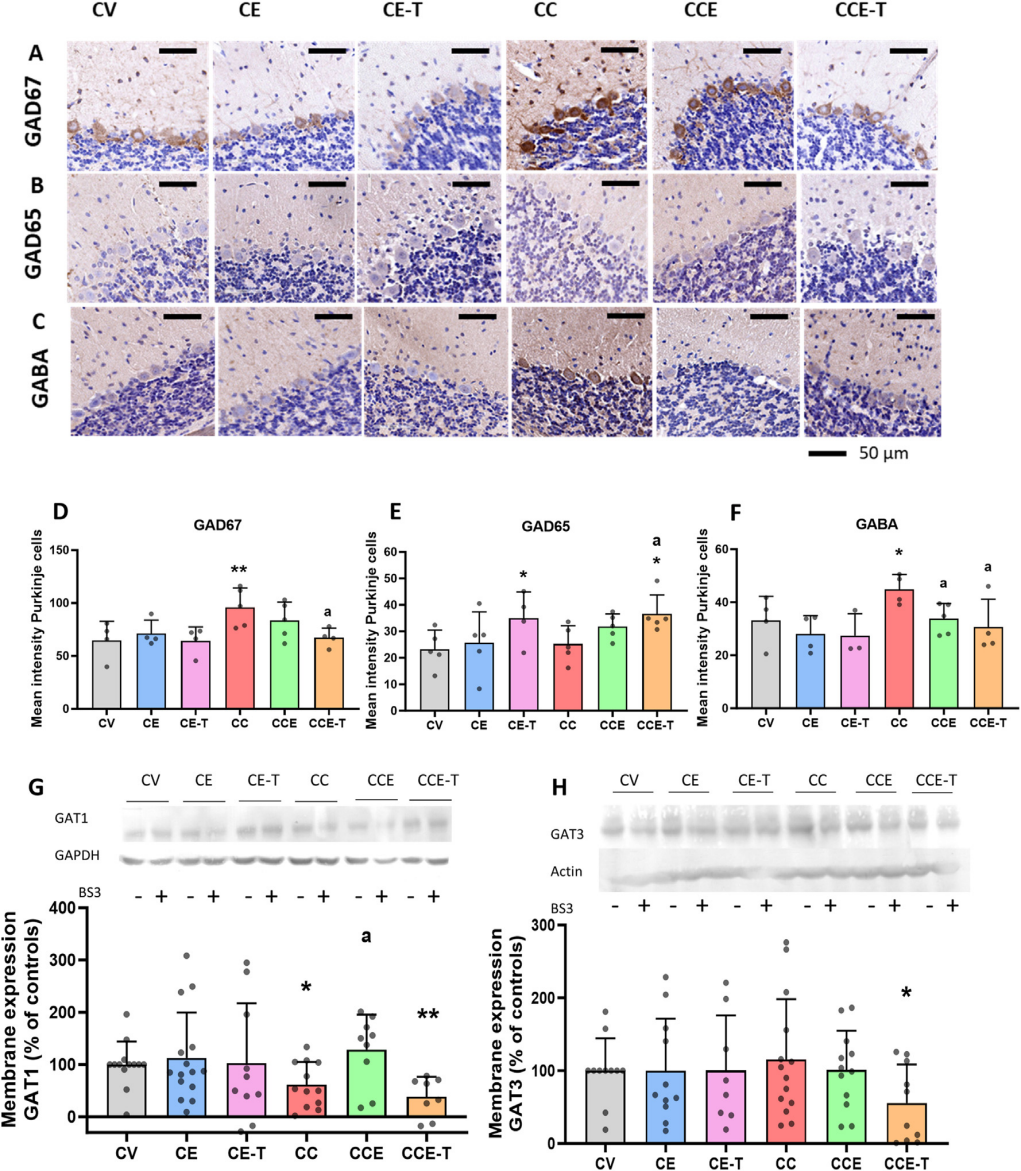
**Effects of MSCs-EVs on GABA and GAD content in PK cells of cerebellum.** Representative images of GAD67 (A) GAD65 (B) and GABA (C) in Purkinje neurons of cerebellum. Values are expressed as of mean intensity of immunostaining and mean ​± ​SD of 3–5 animals per group is represented. Data were analyzed by One-Way ANOVA followed by the Uncorrected Fisher's LSD (D) F ​= ​3.651, p ​= ​0.0371 for T-EVs (no significances for C-EVs) (E) F ​= ​4.725, p ​= ​0.0193 for T-EVs (no significances for C-EVs) (F) C-EVs F ​= ​4.195, p ​= ​0.0279, T-EVs F ​= ​2.938, p ​= ​0.0806. Membrane expression of GABA transporters in cerebellum is shown in G (GAT1) and H (GAT3). Represented data are the mean ​± ​SD of 8–15 rats per group. Data were analyzed with Welch's ANOVA followed by the unpaired t with Welch's correction test, GAT1: C-EVs W ​= ​3.079, p ​= ​0.0478, T-EVs W ​= ​3.982, p ​= ​0.0226; ANOVA for GAT3 was not significant. Asterisks indicate a significant difference compared with CV group ∗p ​< ​0.05, ∗∗p ​< ​0.01, and “a” compared with CC group a p ​< ​0.05. Asterisks indicate a significant difference in the multiple comparison tests compared with CV group ∗p ​< ​0.05, ∗∗p ​< ​0.01, and “a” compared with CC group “a”: p ​< ​0.05.

Impaired motor coordination usually results from altered GABAergic neurotransmission; therefore, we analyzed some key parameters modulating it. A cornerstone factor is the amount of GABA, modulated by enzymes such as glutamate decarboxylases GAD65 and GAD67. The content of both enzymes in Purkinje neurons of the cerebellum was evaluated by immunochemistry. Rats with mild liver failure showed increased content of GAD67 in Purkinje neurons (96 ​± ​8 arbitrary units of intensity (a.u.), p ​= ​0.0095) compared to control rats (65 ​± ​9 a.u) (Fig. 2A,D). GAD67 content was restored to control levels with EVs lacking TGFβ (67 ​± ​4 a.u., p ​= ​0.0149). EVs from MSCs reduced to some extent the content of GAD67 in rats with mild liver damage to 83 ​± ​7 a.u; however, it was not significantly different from CCl_4_ injected rats or from the control group (Fig. 2A,D). The injection of CCl_4_ did not alter the content of GAD65, but treatment with EVs lacking TGFβ increases the content of GAD65 in Purkinje neurons in rats with mild liver damage and in control rats (Fig. 2B,E). Regarding the content of GABA, the rats with mild liver damage showed increased content of GABA in Purkinje neurons (45 ​± ​3 a.u, p ​= ​0.032) compared with control counterparts (33 ​± ​4 a.u.), and treatment with both types of EVs normalized GABA levels (C-EVs 34 ​± ​3 a.u., p ​= ​0.0328; T-EVs 31 ​± ​5 a.u., p ​= ​0.0392) (Fig. 2C,F).

The GABA transporters GAT1 and GAT3 present in the membrane mainly modulate the extracellular concentration of GABA. We analyzed the membrane expression of these transporters. Mild liver damage reduced the membrane expression of GAT1 to around 50% of control rats (p ​= ​0.039, Fig. 2G), which could increase extracellular GABA in the cerebellum. Membrane expression of GAT3 was not altered in CCl_4_-injected rats (Fig. 2H). Treatment with MSC-EVs completely reversed the reduction in membrane expression of GAT1 (p ​= ​0.0218). In contrast, treatment with EVs lacking TGFβ potentiated the reduction of GAT1 in the membrane (p ​= ​0.0036) and significantly reduced membrane expression of GAT3 in rats with mild liver damage (p ​= ​0.0349) (Fig. 2G and H).

### TGFβ-dependent reversion of microglia and astrocyte activation in cerebellum of MHE rats

**Fig. 3:**
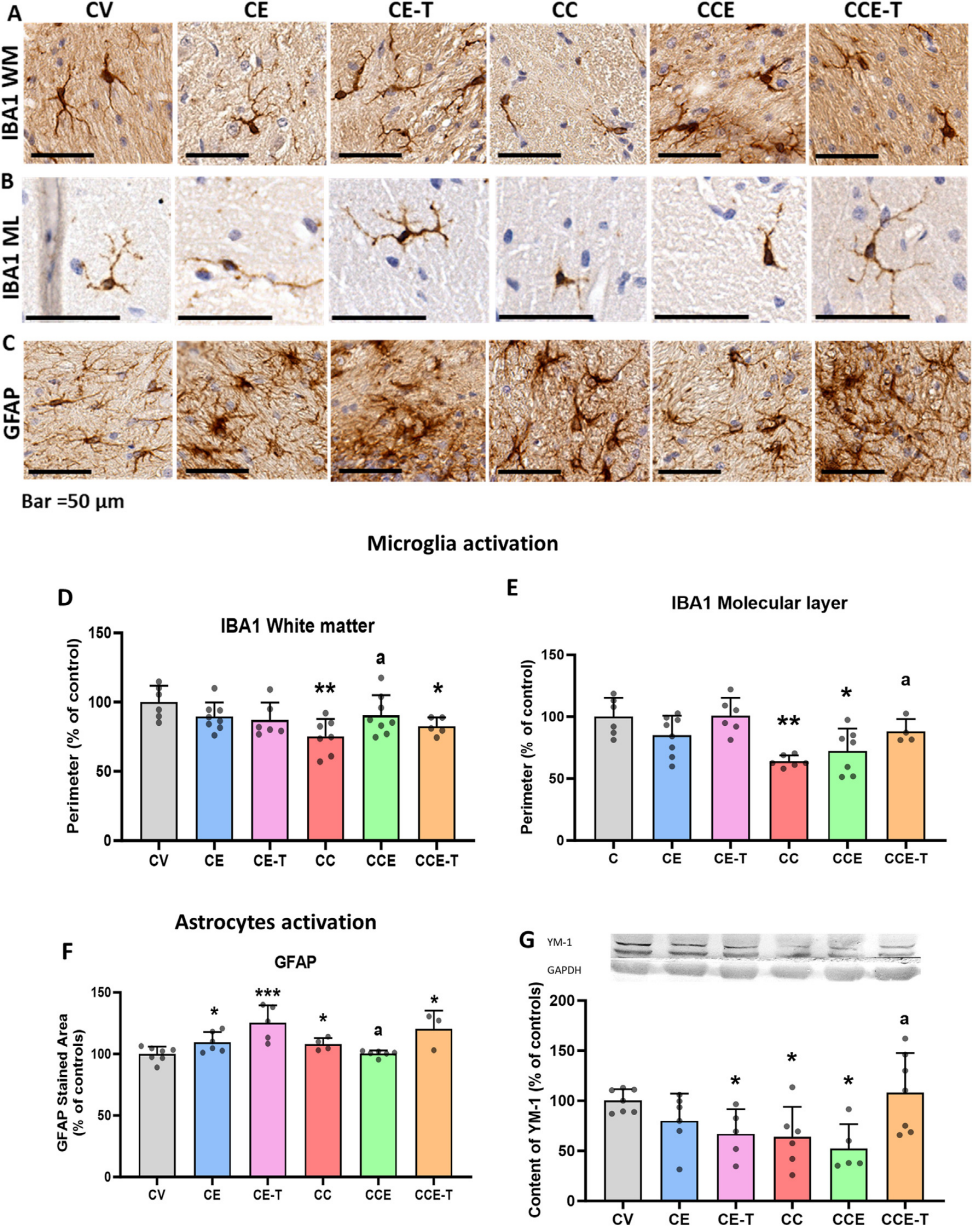
**Effects of MSCs-EVs on microglial and astrocytic activation in cerebellum.** Representative images of microglia marked with Iba-1 in white matter (A) and molecular layer (B) of cerebellum, and astrocytes marked with GFAP in white matter of cerebellum (C). Mean ​± ​SD of 5–8 (D), 4–8 (E) and 3–7 (F) rats per group are represented. Data were analyzed by one-way ANOVA followed by the Uncorrected Fisher's LSD test (D, F) or Tukey's multiple comparisons test (E). (D) C-EVs F ​= ​4.463, p ​= ​0.0121; T-EVs F ​= ​5.235, p ​= ​0.0079; (E) C-EVs F ​= ​6.947 p ​= ​0.0017; T-EVs F ​= ​12.26 p ​= ​0.0001, (F) C-EVs F ​= ​4.257, p ​= ​0.0185; T-EVs F ​= ​6.944, p ​= ​0.0037. (G) YM-1 content in the cerebellum was analyzed by Western blot and is expressed as percentage of control rats. Data are mean ​± ​SD of 5–7 animals per group and were analyzed by one-way ANOVA (F ​= ​3,931, p ​= ​0,0226) followed by the Uncorrected Fisher's LSD test. Asterisks indicate a significant difference compared with CV group ∗p ​< ​0.05, ∗∗p ​< ​0.01, ∗∗∗p ​< ​0.001 and “a” compared with CC group a p ​< ​0.05.

In hyperammonemic rats, enhanced GABAergic neurotransmission in the cerebellum is a consequence of neuroinflammation-induced activation of the TNFα-glutaminase-GAT3 pathway [44] and of the TNFα-CCL2-P2X4-BDNF-TrkB pathway [33,45]. Accordingly, we investigated the effects of mild liver failure and treatment with MSC-EVs on neuroinflammation and these pathways. We observed that MHE rats showed activation of microglia in white matter and the molecular layer of the cerebellum. The perimeter of microglial cells is reduced in white matter to 75 ​± ​5 % (p ​= ​0.0014) (Fig. 3A,D) and in the molecular layer to 64 ​± ​2% (p ​= ​0.0017) (Fig. 3B,E) compared to control rats, indicating activation of microglia. Similarly, MHE rats also showed activation of astrocytes, with increased (p ​= ​0.0463) GFAP stained area (108 ​± ​3 % of controls) (Fig. 3C,F). Given that microglia can be polarized towards pro-inflammatory or anti-inflammatory types, we explored the levels of YM-1 (chitinase-3-like-3), as a marker of anti-inflammatory microglia. The content of YM-1 was reduced in CCl_4_ rats compared to the control rats (64 ​± ​12 % of controls, p ​= ​0.0331) (Fig. 3G). This suggests a higher proportion of pro-inflammatory microglia in rats with mild liver damage. The treatment with MSCs-EVs reversed microglia activation in white matter (Fig. 3A,D) and astrocyte activation (Fig. 3C,F), but it failed to normalize activation of microglia in the molecular layer (Fig. 3B,E) and the decrease of YM-1 (Fig. 3G). In contrast, treatment with MSC-T-EVs reversed the activation of microglia in the molecular layer (Fig. 3B–E) and the decrease in YM-1 (Fig. 3H), but not microglia activation in white matter (Fig. 3A,D) nor astrocyte activation (Fig. 3C,F). Both types of MSCs-EVs induced astrocytic activation in control rats (CE 110 ​± ​3% p ​= ​0.0089, CE-T 125 ​± ​6% p ​= ​0.0007, Fig. 3C, F).

### MSCs-EVs normalize the TNFα-glutaminase-GAT3 and TNFα-CCL2-P2X4-BDNF-TrkB pathways in cerebellum of MHE rats

**Fig. 4:**
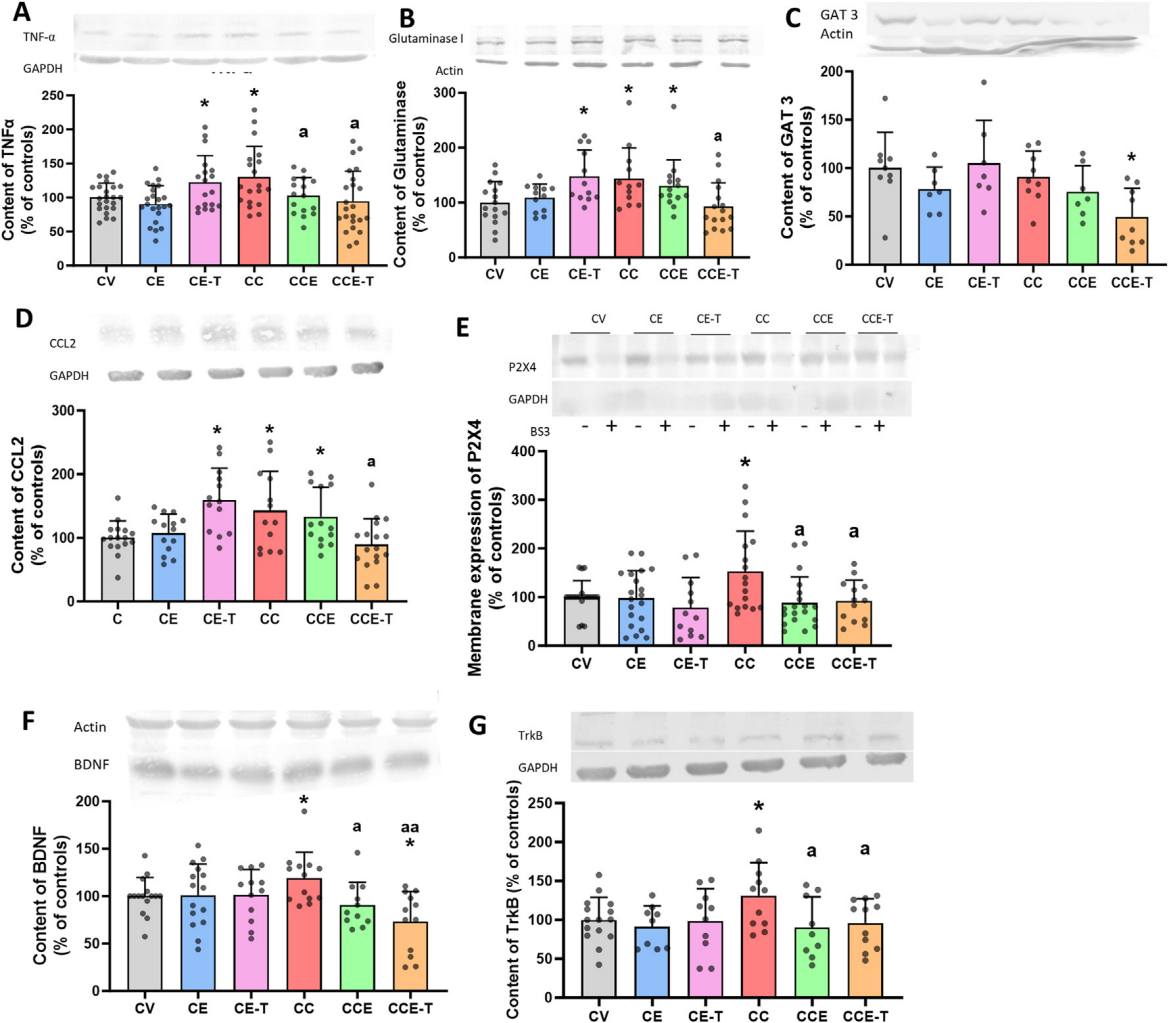
**MSCs-EVs normalize TNF-α, glutaminase and GAT3 content and reduce activation of the TNFα-TNFR1-CCL2-BDNF-TrkB pathway in cerebellum.** Data of content of TNF-α (n ​= ​16–23, A), glutaminase I (n ​= ​12–16, B), GAT-3 (n ​= ​7–9, C) and CCL2 (n ​= ​13–17, D), membrane expression of P2X4 (n ​= ​13–20, E) and content of BDNF (n ​= ​11–15, F) and TrkB (n ​= ​9–15, G) in cerebellum, analyzed by Western blot, are expressed as percentage of controls. Mean ​± ​SD is represented. (A) Data were analyzed with Welch's ANOVA test followed by the Unpaired t with Welch correction test: C-EVs W ​= ​3.588, p ​= ​0.0223; T-EVs W ​= ​3.945, p ​= ​0.0152. (B) one-way ANOVA followed by the Uncorrected Fisher's LSD test (C-EVs F ​= ​2.987, p ​= ​0.0398) and by Tukey's multiple comparisons test (T-EVs F ​= ​5.410; p ​= ​0.0026). (C) one-way ANOVA followed by Tukey's multiple comparisons test (T-EVs F ​= ​4.695, p ​= ​0.0084). (D) Welch's ANOVA followed by the Unpaired t with Welch correction test: C-EVs W ​= ​3.071, p ​= ​0.0445; T-EVs W ​= ​7.207, p ​= ​0.0010. (E) Kruskal-Wallis test (K–W statistic ​= ​7.776, p ​= ​0.0509) followed by uncorrected Dunn's test for C-EVs and Welch's ANOVA followed by Unpaired t with Welch correction test for T-EVs (W ​= ​4.683, p ​= ​0.0068). (F) One-Way ANOVA followed by Uncorrected Fisher's LSD test (T-EVs F ​= ​6.388, p ​= ​0.0010 and C-EVs F ​= ​2.465; p ​= ​0.0731). (G) One-Way ANOVA followed by the Uncorrected Fisher's LSD test (C-EVs F ​= ​3.175, p ​= ​0.0344; T-EVs F ​= ​2.368, p ​= ​0.0839). Asterisks indicate a significant difference compared with CV group ∗p ​< ​0.05, and “a” compared with CC group a p ​< ​0.05, aa p ​< ​0.01.

An enhanced activation of the TNFα-glutaminase-GAT3 pathway mediates a neuroinflammation-induced increase of GABAergic neurotransmission in hyperammonemia [44]. Thus, we sought to see if this circuit is altered in our animal model and whether the MSC-EVs treatments can counteract it. Rats with mild liver damage showed increased content of TNF-α (130 ​± ​10 % of controls, p ​= ​0.0125; Fig. 4A) and glutaminase (144 ​± ​16 % of controls, p ​= ​0.0098) in the cerebellum (Fig. 4B), with no change in the content of GAT3 (Fig. 4C). TNFα levels were normalized by both types of EVs (CCE 103 ​± ​7 % of controls, p ​= ​0.0356; CCE-T 95 ​± ​9 % of controls, p ​= ​0.0137) (Fig. 4A). Glutaminase levels were differentially normalized by MSC-T-EVs (93 ​± ​11 % of controls, p ​= ​0.0258) but not by MSC-EVs (131 ​± ​13 % of controls) (Fig. 4B). MSC-T-EVs increased TNFα (122 ​± ​9 %, p ​= ​0.0391) and glutaminase content (148 ​± ​14%, p ​= ​0.0409) in control rats (Fig. 4A and B) and reduced GAT-3 content (49 ​± ​10% of controls, p ​= ​0.0192) (Fig. 4C). We additionally explored the state of the TNFα-CCL2-BDNF-TrkB pathway, which also modulates GABAergic neurotransmission in the cerebellum [33,45]. Activation of the TNFα receptor TNFR1 increases CCL2, which activates its receptor CCR2 in microglia leading increased membrane expression of P2X4 receptor and of BDNF content that activates its receptor TrkB in the neurons. TrkB activation enhances GABAergic neurotransmission [33]. We then dissect the main components of this pathway and retrieved that in MHE rats there is an increase of TNFα (Fig. 4A), CCL2 (143 ​± ​17 % of controls, p ​= ​0.0327; Fig. 4D), membrane-bound P2X4 (152 ​± ​20 % of controls; Fig. 4E), BDNF (119 ​± ​7 % of controls, p ​= ​0.0428; Fig. 4F) and of its receptor TrkB (131 ​± ​13 % of controls, p ​= ​0.0301; Fig. 4G). Both types of EVs reversed the increase of the membrane expression of P2X4 (Fig. 4E) and the content of BDNF (Fig. 4F) and TrkB (Fig. 4G). The increase of CCL2 was reversed exclusively by MSC-T-EVs (Fig. 4D).

### Components of gut microbiota in MHE rats covary with MSCs-EVs treatments

**Fig. 5:**
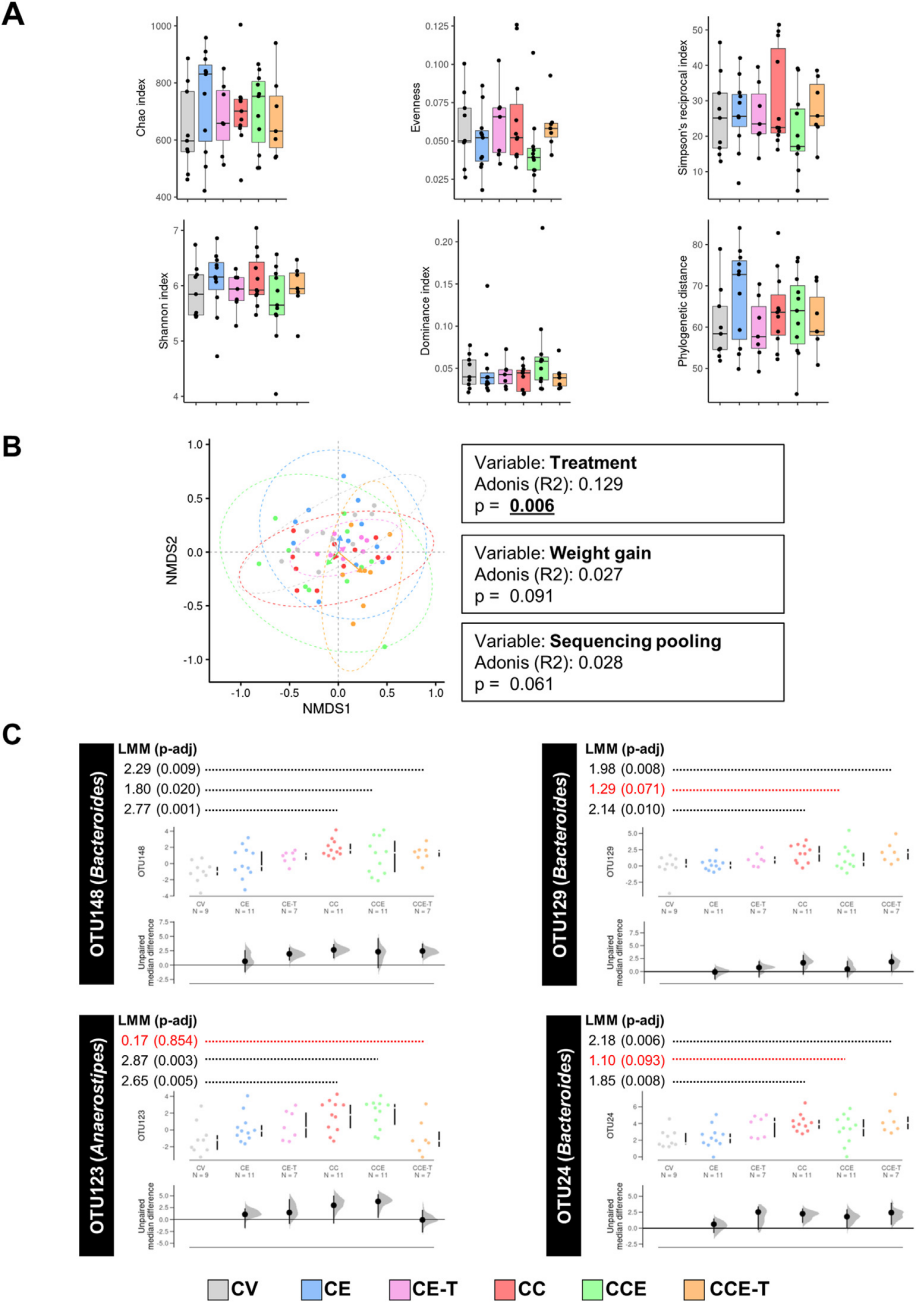
**Microbiota assessment on MHE rats following MSC-EVs treatment. (**A) The alpha diversity assessment including study of the Chao's index, Simpson's evenness index, Simpson's reciprocal index, Shannon index, Dominance index, and phylogenetic distance descriptors. Color legend and group labeling is maintained as in previous figures (see color legend at bottom). Alpha diversity data is presented in a boxplot manner (n ​= ​58 samples) no significantly different data distributions were obtained following Kruskal-Wallis statistical appraisal and Wilcoxon Rank Sum test pairwise comparisons. (B) Beta diversity evaluation of the fecal microbial community structure is provided using distance-based redundancy analysis (dbRDA). The two gradients of dataset dispersion in ordination space explaining more variation of this constrained approach are shown in a scatter-plot fashion. Color, symbols, and labels agree with groups and treatments described in previous panels (see color legend at bottom). Dashed lines circumscribe the confidence interval (95%) for distribution of respective grouped samples. The result of the Adonis test is depicted in the text boxes embedded. Arrows' heads point out the respective centroids of data dispersion. (C) Gardner-Altman estimation plots. Clr-normalized abundance of DNA reads for selected OTUs (*Bacteroides* and *Anaerostipes* species) was processed with *Dabestr* R package to obtain graphics based on shared control analysis, median difference as size effect, and CI 95%. CV data was assumed as reference data. Estimate of LMM score from comparison between control and CEE groups (disease model) and exact adjusted p-values are shown at top of respective panels, respectively. (For interpretation of the references to color in this figure legend, the reader is referred to the Web version of this article.)

As earlier introduced, it has been proposed that changes in the gut microbiome contribute to neuroinflammation in murine cirrhosis [20]. Hence, we assessed the effects of mild liver damage and of treatment with MSC-EVs on gut microbiota. The analysis of individual fecal microbiota composition indicated that none of the alpha diversity descriptors explored showed important shifts across the experimental groups (Fig. 5A). However, when this analysis was achieved at the microbial community structure level (beta diversity), we detected a meaningful different composition among treatment groups. The microbial community composition was mainly explained by the treatment itself (Adonis R2 ​= ​0.129, p ​= ​0.006). The two covariates explored during analysis, weight gain (across the experiment) and sample sequencing pool, exhibited minor impact on the community structures (Fig. 5B). The evaluation of the differential abundance across experimental groups was completed by evaluating the distribution of more than 3500 OTUs. We detected significant signals of differential abundance in almost 200 OTUs, 79 of which were prioritized to have the most prominent variation based on the linear mixed model (LMM) score with covariate control (see methods). To narrow down the list of OTUs to be associated with treatments, we focused on those with reliable taxonomy identification (genus level) and those exhibiting the reversion of changes seen in MHE rats compared to control rats. We detected a predominant presence of OTUs identified as members of the *Bacteroides* genus as well as OTUs classified within the *Intestinimonas*, *Acetatifactor*, *Anaerostipes* and *Muribaculum* genera. To contribute to disambiguating in the *Bacteroides* taxonomy annotation, as well as in other genera detected, we further assessed OTU identity by using SINA aligner (SILVA database) and Blast (NCBI Taxonomy database) automatic servers. We found that *Bacteroides* species likely match with OTU24, OTU129, and OTU148 were *B. eggerthii*, *B. cellulosilyticus*, and *B. caccae*, respectively. Notably, we detected some OTUs showing shifts in their abundance depending of the mild liver damage status and MSC-EVs treatments, suggesting the rat microbiota respond positively to the respective treatments. Particularly, *Bacteroides* OTUs such as OTU148, OTU24, and OTU129 were decreased by MSC-EVs towards abundance seen for the control group (Fig. 5C). On the other hand, the OTU123 (likely *Anaerostipes faecis*) positively responded exclusively to MSC-T- EVs (Fig. 5C). Relative abundance of OTU169 (likely *Intestimonas timonensis)* was increased in MHE rats and it was normalized by both types of EVs.

### Profiling of tryptophan-kynurenine pathway, microbiota metabolites, and TFGβ in MHE rats following MSC-EVs treatments

**Fig. 6:**
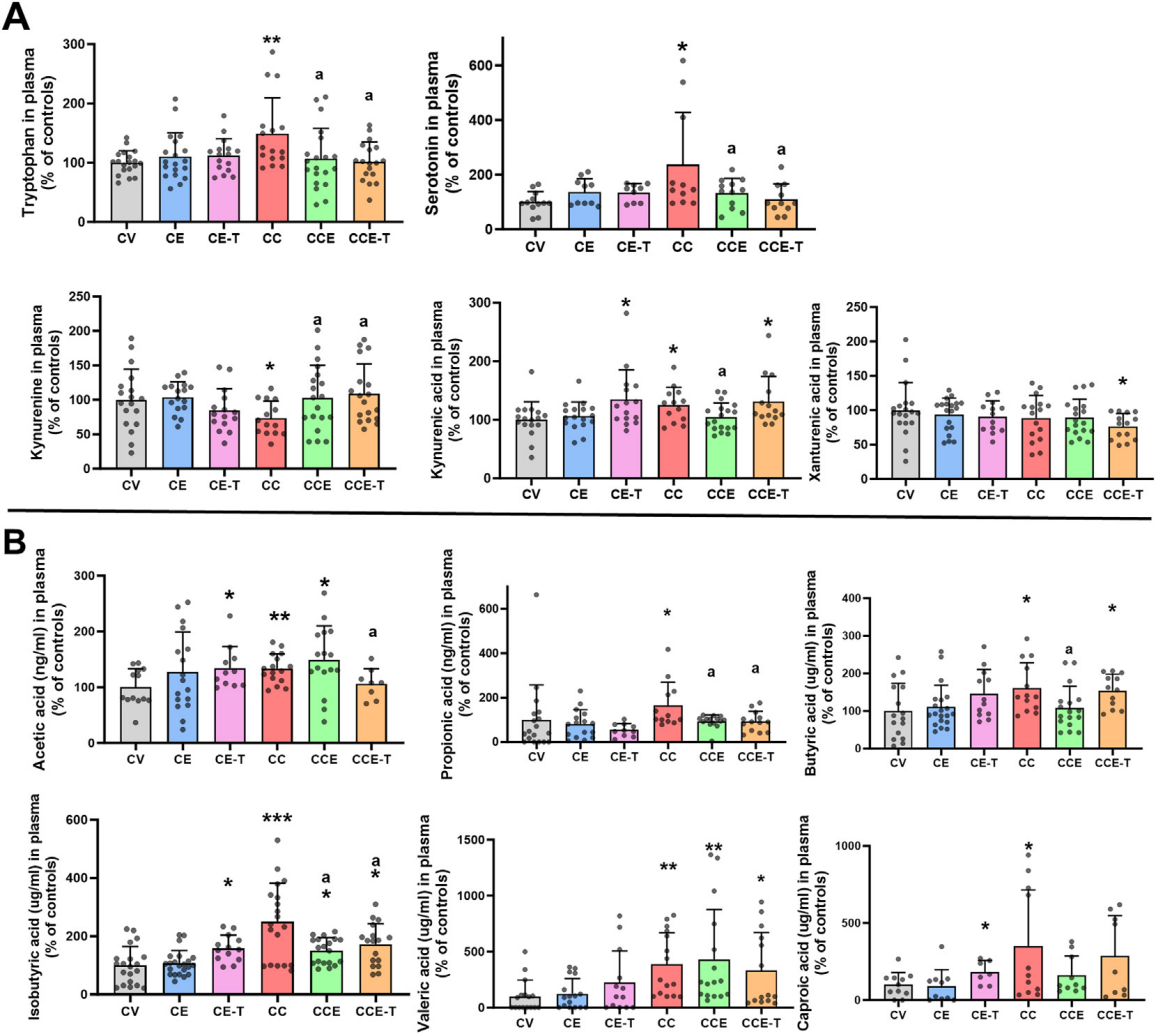
**Plasmatic levels of metabolites of the tryptophan-kynurenine pathway and of SCFAs.** Levels in plasma of tryptophan, serotonin, kynurenine, kynurenic and xanthurenic acid were determined by LC-MS and are expressed as percentage of controls. Data are the Mean ​± ​SD of 7–10 rats per group and 5–6 rats per group for serotonin (A). Plasmatic levels of different SCFAs: acetic, propionic, butyric, isobutyric, valeric and caproic acids, were analyzed by LC-MS and are represented as percentage of controls. Data are the Mean ​± ​SD of 6–10 rats per group (B). A) Data were analyzed with One-way ANOVA followed by Tuckey's test for tryptophan (C-EVs F ​= ​3.981, p ​= ​0.0112 and T-EVs F ​= ​5.946, p ​= ​0.0012), and by Fisher's LSD test for Kynurenine (C-EVs F ​= ​2.158, p ​= ​0.1018 and T-EVs F ​= ​2.772, p ​= ​0.0490), kynurenic acid (C-EVs F ​= ​2.346, p ​= ​0.0816 and T-EVs F ​= ​2.534, p ​= ​0.0662), xanthurenic acid (C-EVs F ​= ​0.5018, p ​= ​0.6823 and T-EVs F ​= ​1.562, p ​= ​0.2084) and serotonin (C-EVs F ​= ​3.736, p ​= ​0.0184 and T-EVs F ​= ​4.081, p ​= ​0.0130, with Tuckey's test). B) Data were analyzed with Kruskal-Wallis followed by Dunn's post hoc test, except were another test is indicated: acetic acid (C-EVs Welch ANOVA, W ​= ​3.657, p ​= ​0,0232 and T-EVs K–W statistic ​= ​8.625, p ​= ​0.0347) propionic acid (Welch ANOVA C-EVs W ​= ​1.934, p ​= ​0.1482 and T-EVs W ​= ​5.200, p ​= ​0.0061), butyric acid (C-EVs K–W statistic ​= ​9.056, p ​= ​0.0286 and T-EVs One-way ANOVA and Fisher's LSD F ​= ​2.755, p ​= ​0.0523) isobutyric acid (C-EVs K–W statistic ​= ​21.83, p ​< ​0.0001 and T-EVs Welch ANOVA W ​= ​8.027, p ​= ​0.0003) valeric acid (C-EVs K–W statistic ​= ​18.64, p ​= ​0.0003 and T-EVs K–W statistic ​= ​12.71, p ​= ​0.0053) caproic acid (C-EVs K–W statistic ​= ​5.012, p ​= ​0.1709 and T-EVs K–W statistic ​= ​3.915, p ​= ​0.2708. Asterisks indicate a significant difference compared with CV group ∗p ​< ​0.05, ∗∗p ​< ​0.01, ∗∗∗p ​< ​0.001 and “a” compared with CC group a p ​< ​0.05.

The interconnection between the brain and gut is well recognized. The intestinal microbiota is pointed out as an essential factor triggering neuroinflammation and associated alterations in neurotransmission and motor coordination and gait via its metabolites disseminated systemically via the bloodstream. Some microbial metabolites involved in this process are short-chain fatty acids (SCFA) and the metabolites related to the tryptophan-kynurenine pathway [46,47]. After inspection of plasma samples, we uncovered that MHE rats showed increased levels of tryptophan (149 ​± ​15 % of controls, p ​= ​0.0115), kynurenic acid (125 ​± ​8 % of controls, p ​= ​0.014), and serotonin (237 ​± ​58% of controls, p ​= ​0.0027); and reduced levels of kynurenine (73 ​± ​7 % of controls, p ​= ​0.0423). MSC-EVs normalized levels of tryptophan (107 ​± ​12 % of controls), kynurenic acid (105 ​± ​6 % of controls), serotonin (133 ​± ​16 % of controls) and kynurenine (103 ​± ​11 % of controls). MSC-T-EVs normalized tryptophan (102 ​± ​8 % of controls), serotonin (110 ​± ​17 % of controls), and kynurenine (109 ​± ​10 % of controls) (Fig. 6A). On the other hand, SCFAs (acetic acid, butyric acid, isobutyric acid, propionic acid, caproic acid and valeric acid) levels were increased in MHE rats (p ​< ​0.05). Both types of EVs reduced levels of propionic and isobutyric acid, whereas MSC-EVs reduced only butyric acid and caproic acid and MSC-T-EVs reduced acetic acid. None of the EVs reduced valeric acid (Fig. 6B). Finally, plasma TGFβ levels were analyzed by ELISA to assess the possible effects of EV treatments with differential TGFβ content. MHE rats showed reduced levels of TGFβ (70 ​± ​5% of controls, p ​= ​0.0467) and MSCs-EVs, irrespective of the TGBβ content, were able to normalize levels of TGFβ (C-EVs to 101 ​± ​11 % of controls, p ​= ​0.0493 and T-EVs to 117 ​± ​16 % of controls, p ​= ​0.0114).

An integrative analysis of microbiota and metabolite data (Table 1) indicated that OTU148 (*B. caccae*) and OTU24 (*B. eggerthii*) correlated negatively with kynurenine and xanturenic acid, respectively (Kendall's tau ​= ​−0.22, p ​= ​0.051 and Kendall's tau ​= ​−0.22, p ​= ​0.057). Besides, OTU129 (*B. cellulosilyticus*) correlated positively with kynurenic acid levels (Kendall's ​= ​0.23, p ​= ​0.042), and OTU123 (*A. faecis*) with valerate levels (Kendall's ​= ​0.45, p ​= ​0.007). Interestingly, the abundance of the three *Bacteroides* OTUs (OTU24, OTU129, OTU148) correlated positively with plasma TGFβ levels across all animal groups (Kendall's tau >0.21, p ​< ​0.03). The analysis of correlations between microbiota abundance and motor function parameters indicated that OTU24 and OTU148 correlated positively with dual stance (Kendall >0.22, p ​< ​0.04), OTU148 also correlated negatively with stride length (Kendall ​= ​−0.25, p ​= ​0.022), and OTU24 also correlated positively with step cycle (Kendall ​= ​0.23, p ​= ​0.033).

**Table 1:** Correlations between the abundance of certain species, plasma levels of some metabolites, and some motor function parameters.

OTU	Correlation with	Kendall's tau	p-value
OTU148	Kynurenine	−0.22	0.051
OTU24	Xanturenic acid	−0.22	0.057
OTU129	Kynurenic acid	0.23	0.042
OTU123	Valeric acid	0.45	0.007
OTU169	Butyric acid	−0.31	0.045
OTU24	TGFβ (plasma)	0.27	0.005
OTU129	TGFβ (plasma)	0.24	0.01
OTU148	TGFβ (plasma)	0.21	0.03
OTU24	Dual stance	0.25	0.02
OTU148	Dual stance	0.23	0.04
OTU148	Stride length	−0.25	0.022
OTU24	Step cycle	0.23	0.033

## Discussion

**Fig. 7:**
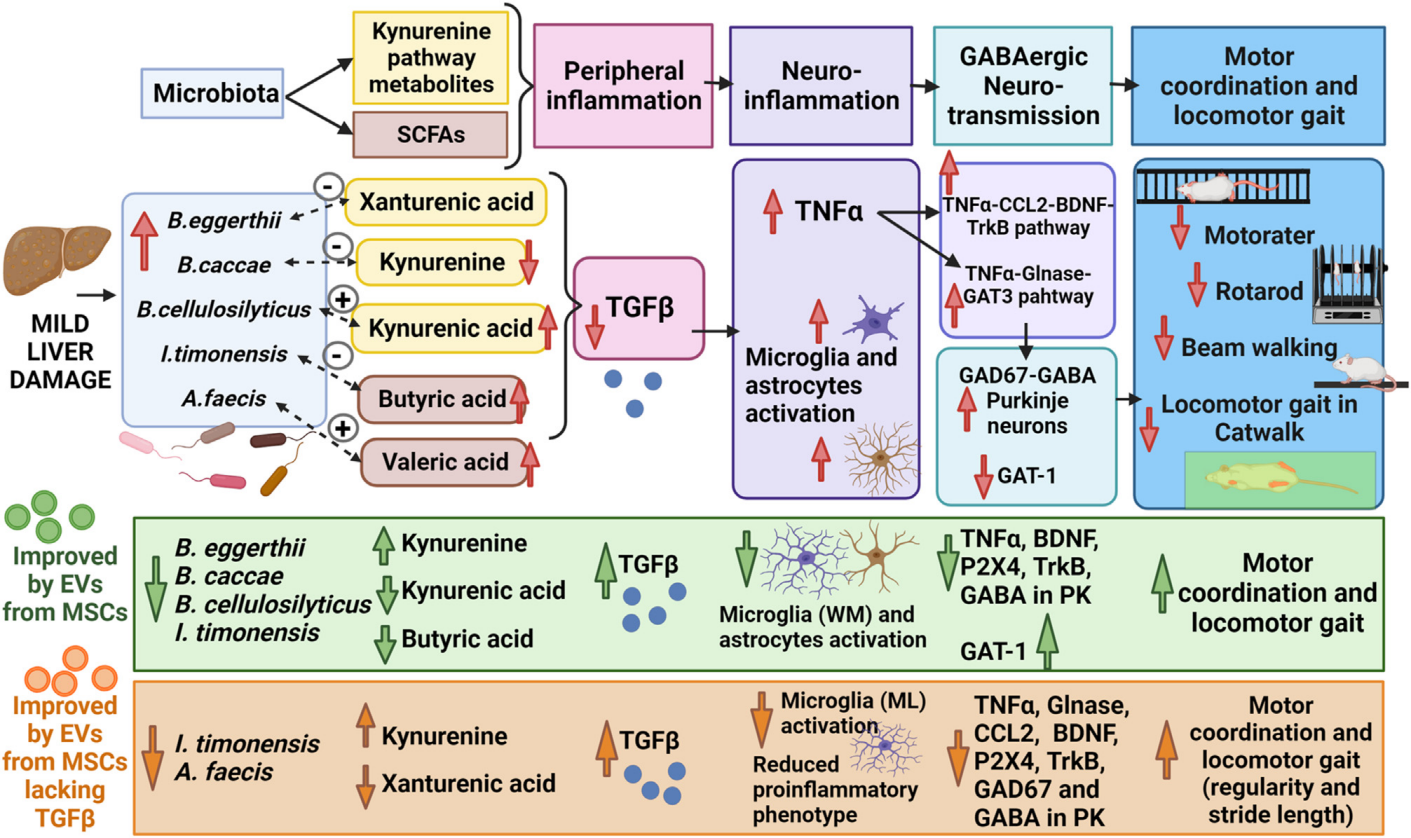
**Proposal of the mechanisms involved in the contribution of dysbiosis to motor impairment in rats with mild liver damage and normalization by MSC-EVs.** Mild liver damage induced by CCl_4_ injection induces dysbiosis, with an increase in different Bacteroides (*B. eggerthii, B. cellulosilyticus,* and *B. caccae), Anaerostipes faecis* and *Intestimonas timonensis*. Dysbiosis is associated with altered plasma content of SCFAs, and of kynurenine and its metabolites, and reduced levels of TGFβ. These peripheral alterations are associated with activation of microglia and astrocytes and increased TNFα levels in the cerebellum, which enhances the activation of the TNFα-CCL2-BDNF-TrkB and TNFα-glutaminase-GAT3 pathways. This is associated with enhanced GABAergic neurotransmission, with increased levels of GAD67 and GABA in Purkinje neurons and reduced membrane expression of the GABA transporter GAT1, which would increase extracellular GABA. Enhanced GABAergic neurotransmission leads to impaired motor coordination and gait. Treatment with MSC-EVs reverses motor incoordination and alterations in locomotor gait, but the mechanisms involved are different for normal EVs and for EVs lacking TGFβ. Treatment with normal MSC-EVs reverse the increase in Bacteroides and *I. timonensis* and normalizes the plasma levels of butyrate, kynurenine, kynurenic acid and TGFβ. These EVs also reverse microglia activation in white matter but not in the molecular layer, astrocyte activation and the activation of the TNFα-CCL2-BDNF-TrkB pathway. This is associated with normalization of GABA in Purkinje neurons and of membrane expression of GAT1 as well as of motor coordination and of all the parameters altered in locomotor gait in the Catwalk. Treatment with MSC-EVs lacking TGFβ affects differently microbiota, reversing the increase in *I. timonensis* and *A. faecis*, but not in Bacteroides. They also reverse changes in plasma kynurenine and TGFβ but not in kynurenic acid or butyrate. Concerning neuroinflammation, MSC-EVs lacking TGFβ reverse microglia activation in molecular layer, but not in white matter nor astrocyte activation. They induce a shift of microglia to an anti-inflammatory state. This is associated with reversal of changes in GABAergic neurotransmission, with normalization of GAD67 and GABA in Purkinje neurons, and of motor incoordination, and of some, but not all parameters of locomotor gait. Red arrows indicate increase or decrease in rats with mild liver damage. Green arrows indicate steps normalized by C-EVs and orange arrows indicate normalization by T-EVs. Discontinued arrows indicate correlations. (For interpretation of the references to color in this figure legend, the reader is referred to the Web version of this article.)

The global results obtained in our study are graphically reported and summarized in Fig. 7. MHE induced by CCl_4_ injection induces alteration of the gut microbiota, with an increase in different *Bacteroides* species (*B. eggerthii*, *B. cellulosilyticus*, and *B. caccae*), as well as in *A. faecis* and *I. timonensis*. Such shifts in microbial abundance are associated with altered plasma content of SCFAs, including increased butyrate levels, altered kynurenine and kynurenic acid content, and reduced levels of TGFβ. These peripheral alterations are associated with neuroinflammation in the cerebellum, with activation of microglia and astrocytes and increased TNFα levels, which enhance the activation of the TNFα-CCL2-BDNF-TrkB and TNFα-glutaminase-GAT3 pathways. This is associated with enhanced GABAergic neurotransmission, with increased levels of GAD67 and GABA in Purkinje neurons and reduced membrane expression of the GABA transporter GAT1, which would increase extracellular GABA. Enhanced GABAergic neurotransmission leads to impaired motor coordination and gait. In addition, serotonin levels in plasma were also increased in CCl_4_ rats.

Treatment of MHE rats with MSC-EVs reverses motor incoordination and alterations in locomotor gait. However, the mechanisms involved seem to be are different for MSC-EVs and MSC-T-EVs (Fig. 7). Treatment with normal MSC-EVs reverse the increase in *Bacteroides* and *I. timonensis* and normalizes the plasma levels of butyrate, kynurenine, kynurenic acid, serotonin and TGFβ. These EVs also reverse microglia activation in white matter but not in the molecular layer, astrocyte activation and the activation of the TNFα-CCL2-BDNF-TrkB pathway. Such effect is associated with normalization of GABA in Purkinje neurons and of membrane expression of GAT1 as well as of motor coordination and all the parameters altered in locomotor gait in the Catwalk (Fig. 7). Besides, treatment with MSC-T-EVs affects differently microbiota, reversing the increase in *I. timonensis* and *A. faecis*, but not in *Bacteroides*. They also reverse changes in plasma kynurenine, serotonin and TGFβ but not in kynurenic acid or butyrate. Concerning neuroinflammation, MSC-EVs lacking TGFβ reverse microglia activation in molecular layer, but not in white matter nor astrocyte activation. They induce a shift of microglia to an anti-inflammatory state, which is associated with reversal of changes in GABAergic neurotransmission, with normalization of GAD67 and GABA in Purkinje neurons motor incoordination, and some parameters of locomotor gait (Fig. 7).

The role of TGFβ present in the MSC-EVs in the anti-inflammatory effects of these EVs has been previously reported. In a mouse model of sepsis MSC-EVs alleviated sepsis-induced acute liver injury, reduce inflammation, induce TGF-β secretion in macrophages and increase regulatory T cells (Tregs). TGF-β silencing reversed these alleviating effects of MSC-EVs [48]. Injection of MSC-EVs to hyperammonemic rats improves cognitive and motor function in parallel with improvement of neuroinflammation and alterations in neurotransmission in the hippocampus and cerebellum [30]. The addition of MSC-EVs *ex vivo* to hippocampal or cerebellar slices from hyperammonemic rats also improved neuroinflammation and neurotransmission. These beneficial effects were mediated by TGFβ, and were lost when TGFβ action was blocked. Here, we show that, in rats with mild liver damage induced by CCl_4_ injection, both MSC-EVs and MSC-T-EVs reduce neuroinflammation and reverse alterations in GABAergic neurotransmission in the cerebellum and improve motor function. However, that occurs through different mechanisms, thus paving the way to find novel EVs compounds with similar function than TGFβ.

Cirrhotic patients with MHE or clinical HE show impaired motor coordination and locomotor gait [[49], [50], [51], [52], [53]]. We showed that our animal model, mimicking MHE phenotype via CCl_4_ injection, also shows motor incoordination and altered locomotor gait. Interestingly, motor incoordination was improved by treatment with MSCs-EVs regardless of the TGFβ presence, suggesting that MSCs-EVs can improve motor incoordination by mechanisms which do not involve a direct action of this key player of the immune response. Concerning locomotor gait, MHE rats showed alterations in dual stance, step cycle, stride length, regularity index and stand index. Reduced stride length and increased dual stance are characteristic alterations of locomotor gait in cerebellar ataxia [54]. Moreover, increased dual stance, reduced stride length, and increased stand index and step cycle have been related to bradykinesia and action-start hesitation symptoms in Parkinson's disease patients [55,56]. Bradykinesia and impairment of movement initiation are also common symptoms in patients with MHE [53,57]. Our results support the notion that treatment with normal MSC-EVs reversed all the altered gait parameters, while EVs lacking TGFβ did not improve dual stance, step cycle or stand index, strongly suggesting a central role of TGFβ on the amelioration of motor function in MHE. Similar beneficial effects were already reported in a mouse model of Parkinson's disease [58]. In the same line of thoughts, dual stance and step cycle, which were normalized by unmodified EVs but not by EVs lacking TGFβ, are related to freezing of gait, a typical symptom in Parkinson's patients [56]. Previous preclinical reports describe improved motor coordination and gait parameters in a rat model of Parkinson's disease following intranasal administration of EVs derived from human teeth stem cells [59]. The results reported here suggest that distinct mechanisms modulate different gait parameters, some being TGFβ dispensable.

To shed light on the probable underlying mechanisms of MSC-EVs action seen here, we took advantage of previous insights indicating that motor incoordination is usually due to enhanced GABAergic neurotransmission in the cerebellum [60] to which enhanced activation of the TNFα-glutaminase-GAT3 and the TNFα-CCL2-BDNF-TrkB pathways are main contributors [33,44]. We have shown that these two pathways are altered in the cerebellum of MHE rats, which also show increased GAD67 and GABA in Purkinje neurons and reduced GAT1 levels. All these changes enhance GABAergic neurotransmission in the cerebellum and induce motor incoordination. Both types of MSC-EVs reverse the alterations in the two above pathways and on GABA in Purkinje neurons. However, both types of tested EVs affect differentially GABA transporters, pointing out an interaction between GAT-1 and TGFβ, and explaining the slight differences in normalization of locomotor gait by both EV types.

The alterations in GABAergic neurotransmission derive from neuroinflammation in the cerebellum; thus, reducing neuroinflammation restores GABAergic neurotransmission and improves motor coordination [8,9]. Our group has previously demonstrated that locomotor gait alterations are also reversed by reducing neuroinflammation by blocking S1PR2 [33] or with golexanolone, which acts on GABA_A_ receptors and reduces neuroinflammation [61]. Here, we report that MSC-EVs are also able to reduce neuroinflammation via attenuation of astrocyte and microglia activation in the white matter of the cerebellum, which would restore GAT1 membrane expression, decrease extracellular GABA levels and improve motor incoordination. The inhibition of microglia activation and improvement of motor performance by TGFβ has been previously reported in a model of Parkinson's disease [62]. In this line of thought, our results corroborate such early observations, given MSC-EVs lacking TGFβ did not improve astrocyte or microglia activation in white matter. Nevertheless, they did improve microglia activation in the molecular layer of the cerebellum, hence there seems to exist a different spatial scope between both types of EVs [63]. In addition, MSC-T-EVs change the microglia phenotype towards an anti-inflammatory state, which must contribute to reduce TNFα and to the beneficial effect of TGFβ on GABAergic neurotransmission and motor function. Such positive effect resembles the sulforaphane or sildenafil action, which also promote polarization of microglia to the anti-inflammatory phenotype, increasing YM-1 in the cerebellum of MHE rats, reducing TNFα and normalizing extracellular GABA, learning, and motor coordination [64,65].

The microbiome research conducted in the latest years unequivocally indicates that microbiota-gut-brain axis has a role in the modulation of neuroinflammation and brain function. Perturbation of the gut microbiota can activate enteric immune cells and promote the release of inflammatory factors and metabolites triggering neuroinflammation [46]. In chronic liver disease, the shift of gut microbiota profiles increases intestinal permeability resulting in translocation of bacteria and endotoxins, which may contribute to hepatic encephalopathy development [66]. We disclosed that MHE rats show an altered composition of the gut microbiota, with increased abundance of certain *Bacteroides* species *(B. eggerthii, B. cellulosilyticus,* and *B. caccae),* and *Anaerostipes* and *Intestimonas* associated OTUs. Larger proportion of *Bacteroides* species has been reported in patients with non-alcoholic steatohepatitis [67], this observation suggests that our rat model reproduces the alterations present in such patients. Moreover, we found that the abundance of these species correlates with plasma levels of some microbiota metabolites, TGFβ levels, and with some parameters of locomotor gait altered in rats with mild liver damage. Although the above described is not enough evidence for causality, it does serve as starting point for future investigations given the intricate covariation and correlation depicted between specific microbiota components and physiological parameters explored across all experimental groups. Consequently, we found that MSC-EVs treatment reduces *B. cellulosilyticus, B.caccae*, *B.eggerthii* and *I. timonensis* while MSC-T-EVs normalized *I. timonensis* and *A. faecis*. MSC-EVs have been reported to modulate gut microbiota in other pathologies. For instance, Yan et al. [68] reported in mice that MSC-EVs reach the intestine and remain there 7 days after MSC-EVs injection. They proposed that MSC-EVs reduce inflammation by regulating the Treg/Th17 ​cells balance, reversing colon epithelium alterations and modulating microbiota composition towards promoting expansion of beneficial species. Intriguingly, the therapeutic effects of MSC-EVs are thought to depend on microbiota composition and derived metabolites. In a mouse model of Alzheimer disease, microbiota depletion increased tryptophan and kynurenic acid levels and enhanced the beneficial effects of MSC-EVs [69]. Therefore, our observations on the correlations among gut microbiota abundance, metabolites and phenotypes presented here are promising to establish MSC-EVs mechanisms of action in the future.

It is noteworthy that MSC-EVs with and without TGFβ modulate the abundance of different types of bacteria. This suggests that TGFβ contributes to the modulation of the gut microbiota. Such a role has been described in mutant mice with deficient TGFβ signaling, which show an altered gut microbiota with decreased *Bacteroides* species [70]. MHE rats show reduced plasma TGFβ and increased *Bacteroides* species in the gut. Both changes are reversed by MSC-EVs containing TGFβ while EVs lacking TGFβ did not reverse those *Bacteroides* species proportions. Although the mechanisms involved remain unclear, the above reports support that MSC-EVs and TGFβ present in them modulate the composition of gut microbiota and may have implications for reversing microbiota changes in certain pathologies. Moreover, the apparent disparate association between TGFβ and *Bacteroides* species derived from previous studies and our results can be explained by the complexity of this particular bacterial taxon, and the limitation of short-read length technology to permit taxonomy surveys at species level.

The effects of the gut microbiota alteration on inflammation, neuroinflammation and behavior are mainly mediated by bacteria-derived metabolites, such as serotonin, SCFAs and metabolites of the tryptophan-kynurenine pathway [47,71,72]. Also, SCFAs have been described to modulate liver steatosis and inflammation [67]. In a germ-free animal model, a reduction of serotonin production in the gut (enterochromaffin cells in the gut are major producers of serotonin in the organisms, mediated by tryptophan hydroxylase) along with reduction of plasma levels was reported [72]. This supports that increased plasma serotonin in CCl_4_ rats could be due to altered microbiota, although no correlation has been found. An alteration of brain serotonergic system has been reported in different animal models of hepatic encephalopathy, including rats with bile-duct ligation [73,74]. An increase in serotonin synthesis was reported in CCl_4_-induced cirrhotic rats [75]. Serotonergic afferents in the rat cerebellum are mainly located in the molecular layer and close to Purkinje neurons [76]. They modulate glutamatergic and GABAergic neurotransmission in this area and motor function [[76], [77], [78], [79]].

In addition, modulation of gut microbiota affects brain serotonin levels [72]. Microbiota produces serotonin and changes of serotonin levels in the gut and blood circulation could be transmitted to the brain. This may be regulated by the enteroendocrine system, through the vagus nerve or by crossing the blood-brain barrier (BBB) [71], which is disrupted in the mild liver damage rat model used in this work [8]. Under these conditions, not only serotonin, but all gut microbiota metabolites (SCFAs, kynurenine pathway metabolites, bile acids, …) can reach the nervous system. Another pathway contributing to the effects of microbiota alterations on brain is the modulation of the immune system. For example, SCFAs modulate infiltration of peripheral blood monocytes into the brain and activation of microglia by TNF-α [71], which are events occurring in the cerebellum of these rats with CCl_4_-induced mild liver damage [8].

*Bacteroides eggerthii* species induce metabolism of tryptophan towards the production of indole-3-acetic acid [80]. The increase of *B. eggerthii* in rats with mild liver damage may contribute to the reduction of kynurenine in plasma, by increasing tryptophan metabolism to indole-3-acetic acid and reducing its metabolism to kynurenine. The reduction of the *B. eggerthii* abundance by MSC-EVs would contribute to the increase in kynurenine. Moreover, kynurenine inhibits the activation of microglia by LPS [81]; then, reduced kynurenine levels may contribute to increased microglia activation in cerebellum in MHE rats while the increase of kynurenine by MSC-EVs may contribute to the reduction of microglia activation in these animals.

In summary, this works shows that in an animal model of MHE, MSC-EVs treatment can restore gut microbiota changes, neuroinflammation and alterations in GABAergic neurotransmission in the cerebellum, leading to improvement of motor impairment. Both MSC-EVs and MSC-T-EVs induce these beneficial effects, but the underlying mechanisms are different. The data suggest a possible mechanism by which altered microbiota in rats with liver damage may contribute to neuroinflammation and motor impairment due to the increased abundance of certain *Bacteroides* species which would reduce plasma kynurenine thus enhancing microglia activation and neuroinflammation. MSC-EVs injection normalizes *Bacteroides* abundance, kynurenine levels, microglia activation and neuroinflammation, thus restoring motor function. Our results support the therapeutic value of MSC-EVs for translational approaches to treating steatotic liver disease or cirrhotic patients with altered motor function, and also for other diseases in which altered gut-brain axis contributes to motor dysfunction.

## Data sharing

The raw amplicons sequencing data generated from gut microbiota analysis can be accessed through the European Nucleotide Archive (ENA) via bioproject PRJEB71610.

All other data generated in this study are available from the corresponding author upon request.

## Author Contributions

**G.M.:** investigation (performed experiments, data analysis and presentation) and writing **V.F.:** conceptualization, supervision, writing, project administration and funding **V. M-M.:** resources **A.B-P.:** investigation, data curation, formal analysis, visualization and writing **M.L.:** formal analysis, validation, visualization, supervision and writing.

## Declaration of competing interest

The authors declare that they have no known competing financial interests or personal relationships that could have appeared to influence the work reported in this paper.
